# IMPACT OF A KINESIOLOGY COURSE ON EXPECTATIONS REGARDING AGING AND AGEIST BEHAVIORS

**DOI:** 10.1093/geroni/igae098.0048

**Published:** 2024-12-31

**Authors:** Kate Hamel

**Affiliations:** San Francisco State University, San Francisco, California, United States

## Abstract

Students majoring in Kinesiology often aspire to work in healthcare or fitness professions where they will frequently work with older adults. Many students don’t recognize this opportunity however, and are often initially drawn to Kinesiology through their own experience with exercise or as athletes and generally assume that they will work with a young, active population in their future careers. Younger adults have also been shown to have negative perceptions and attitudes toward aging and this may shape the way they interact with older adults and could impact their career choice. The purpose of this study was to examine the expectations regarding aging (ERA) and the prevalence of ageist behaviors in students taking a Kinesiology elective course on aging, prior to and after the completion of the course. Students taking the course in two different semesters completed the ERA-12, the Relating to Older People Evaluation and open-ended questions to assess their perceptions, attitudes and behaviors toward aging and older adults. No differences were found between the two cohorts of students in any of the baseline or end of semester measures. Following the completion of the course, students had significantly higher ERA-12 scores and expectations regarding physical health, cognitive functioning and mental health in older adults all improved. There was no difference in positive ageist behaviors, but there was a reduction in negative ageist behaviors. These findings suggest that course-based interventions such as this one, may significantly improve attitudes toward aging and increase student’s interest in working with older adults.

